# Molecular basis of African yam domestication: analyses of selection point to root development, starch biosynthesis, and photosynthesis related genes

**DOI:** 10.1186/s12864-017-4143-2

**Published:** 2017-10-12

**Authors:** Roland Akakpo, Nora Scarcelli, Hana Chaïr, Alexandre Dansi, Gustave Djedatin, Anne-Céline Thuillet, Bénédicte Rhoné, Olivier François, Karine Alix, Yves Vigouroux

**Affiliations:** 1 Institut de Recherche pour le Développement, Université de Montpellier, Unité Mixte de Recherche Diversité Adaptation et Développement des Plantes (UMR DIADE), 911, avenue Agropolis, 34394 Montpellier, France; 2 Unité Mixte de Recherche Génétique Quantitative et Evolutive – Le Moulon, INRA – Univ. Paris-Sud – CNRS – AgroParisTech, Université Paris-Saclay, 91190 Gif-sur-Yvette, France; 3 Faculté des Sciences et Techniques de Dassa, Laboratoire de Biotechnologie, Ressources Génétiques et Amélioration des Espèces Animales et Végétales (BIORAVE), Université d’Abomey, Dassa-Zoumè, Benin; 4 Centre International de la Recherche Agronomique pour le Développement, UMR AGAP, F-34398 Montpellier, France; 5 Université Lyon 1, CNRS, UMR 5558, Laboratoire de Biométrie et Biologie Evolutive, Lyon, France; 6 Université de Grenoble, Grenoble, France

**Keywords:** Domestication, *Dioscorea spp.*, Adaptation, Population genomics, selection, Root development, Starch biosynthesis, Plant development

## Abstract

**Background:**

After cereals, root and tuber crops are the main source of starch in the human diet. Starch biosynthesis was certainly a significant target for selection during the domestication of these crops. But domestication of these root and tubers crops is also associated with gigantism of storage organs and changes of habitat.

**Results:**

We studied here, the molecular basis of domestication in African yam, *Dioscorea rotundata*. The genomic diversity in the cultivated species is roughly 30% less important than its wild relatives. Two percent of all the genes studied showed evidences of selection. Two genes associated with the earliest stages of starch biosynthesis and storage, the sucrose synthase 4 and the sucrose-phosphate synthase 1 showed evidence of selection. An adventitious root development gene, a *SCARECROW-LIKE* gene was also selected during yam domestication. Significant selection for genes associated with photosynthesis and phototropism were associated with wild to cultivated change of habitat. If the wild species grow as vines in the shade of their tree tutors, cultivated yam grows in full light in open fields.

**Conclusions:**

Major rewiring of aerial development and adaptation for efficient photosynthesis in full light characterized yam domestication.

**Electronic supplementary material:**

The online version of this article (10.1186/s12864-017-4143-2) contains supplementary material, which is available to authorized users.

## Background

One of the major changes in human history was the emergence of agricultural societies [1]. About 13,000 years ago, farmers began to domesticated plants and animals for agriculture. Domestication was done by selecting plants and animals with suitable traits for farming like increased yield. As a result, the morphology of our cultivated plants was reshaped by human selection for a period certainly spanning thousands of years [2–4]. The domestication process offers an interesting glimpse of the broad adaptation process and of the genetic basis of morphological and physiological traits [5, 6]. It helps understand how a relatively lowly productive wild relative can be transformed into a high yielding cultivated variety. Insights into crop domestication have primarily come from cereals [5]. Root and tuber crops are also a major contributor of starch to the human diet. These crops have the particularity of very often being vegetatively propagated [7]. The domestication process increased their ability to store starch in their roots or tubers and other specialized storage organs as well as the size of these organs [7]. Today it is not clear if the knowledge we have of the process of domestication of cereal crops can be extrapolated to root and tuber crops. For example, selection on several genes responsible for starch biosynthesis has been documented in maize [8, 9]. So, one would expect that domestication also allows more efficient production and/or storage of starch in root and tuber crops. One would also expect that domestication reshaped the formation and development of roots as a support for efficient starch storage.

The most widely grown root and tuber crops in Africa are cassava and yam. The two main species of yam, *Dioscorea spp*., were domesticated independently, *D. rotundata* in Africa and *D. alata* in Asia. *D. rotundata*, the most widely cultivated yam species in Africa is a staple food for over 100 million people [10]. This species has two close wild relatives *D. abyssinica* and *D. praehensilis* [11–14]. The three species are diploid and have 20 chromosomes [2n = 40] [14–16]. The African cultivated yam and its closest wild relatives are compulsory out-crossers because they are dioecious. However, *D. rotundata* is preferentially propagated through vegetative multiplication [17]. Interestingly, the two wild species have distinct ecological distribution: *D. abyssinica* is found in the wooded savanna areas while *D. praehensilis* is found in tropical forested areas [18]. The diploid African yam is cultivated in both ecological areas, thereby allowing gene flow between cultivated and the two wild species [13]. Several key phenotypes differentiate cultivated varieties from their wild relatives. Cultivated yams are characterized by larger and less ramified roots than their wild relatives, and some cultivated varieties do not develop inflorescences [19]. Finally, the wild relatives of yam are vines which grow partly in the shade of their tutor tree, while cultivated yams grow in full sunlight. This change of habitat might be associated with major adaptation.

Our objective was to uncover the molecular basis of yam domestication. To find what genes and specific functions were selected during yam domestication, we sequenced the genome of wild and cultivated African yams. Using this dataset, we then scanned for selection signature to pinpoint genes associated with domestication.

## Methods

### Plant material and DNA sequencing

Thirty plants were collected in 15 villages in Benin (Additional file 2: Table S1). Sampling included 10 individuals belonging to the cultivated species *D. rotundata,* and 10 individuals belonging to each of its two closest wild relatives*, D. abyssinica* and *D. praehensilis*. Plants were identified by Serge Tostain (yam specialist, IRD), Nora Scarcelli (yam specialist, IRD) and local yam farmers. DNA was extracted as previously described using a standard protocol [16]. Genomic libraries were constructed using a recent protocol [20]. The genomic libraries were 2 × 100 bp paired-end sequenced by sample multiplexing using the Illumina HiSeq 2000 technology (GeT_Genotoul, Toulouse, France).

### Bioinformatics analysis and SNP detection

Raw data were first filtered using a previously described pipeline [21]. Briefly, we performed a demultiplexing python script demuladapt (https://github.com/Maillol/demultadapt). Adaptors and low-quality bases were eliminated using cutadapt 1.2.1 [22]. Reads with a mean quality score < 30 were removed using a free perl script https://github.com/SouthGreenPlatform/arcad-hts/blob/master/scripts/arcad_hts_2_Filter_Fastq_On_Mean_Quality.pl . Mapping was performed using default options of BWA aln-sampe V0.7.5a–r405 [23], and using the *D. rotundata* transcriptome reference [24]. We validated by modelling that the mapping of genomic DNA reads on a transcriptome reference did not lead to major bias of SNP identification (Additional file 1: Table S1).

We estimated the genotype likelihood (GL) for each site using the option “-GL 3” (SOAPsnp model) implemented in angsd 0.700 [25]. We also performed SNP calling using the HaplotypeCaller in the Genome Analysis Toolkit (GATK) V-3.4-46 [26]. Default options of GATK and the “-rf BadCigar” options were used. SNPs were filtered for low missing rate < 5% and a mean depth ≥ 4. The complete script from the raw data to the GL or SNP data analysis is available as a Additional file 1: Table S1.

### Analysis of diversity, population structure and linkage disequilibrium

Genetic structure was assessed using a least-squares optimization approach implemented in the sNMF program [27]. This approach is based on SNP calling and consists in estimating admixture coefficients based on sparse non-negative matrix factorization [27]. We assessed a number of K populations varying from 1 to 6 clusters. Ten replications were performed for each K value. To select the best K value, we used the minimum value of the cross entropy criterion [27]. We also used the maximum likelihood structure approach implemented in the NgsAdmix program [28]. This approach directly uses the genotype likelihood given by angsd, without calling genotypes. The most relevant K number of population was selected by comparing the results obtained with NgsAdmix and sNMF. Genetic diversity was estimated using nucleotide diversity π [29] and nucleotide polymorphism θ [30] computed using the option “-doThetas” implemented in angsd 0.700 [31]. We calculated the ratio of diversity between the cultivated species *D. rotundata* and each of the wild species *D. praehensilis* and *D. abyssinica* using the R package. Pairwise linkage disequilibrium (LD) was calculated with the squared allele frequency correlation *r* ^2^ [32] using the R packages SNPRelate [33] and LDcorSV [34]. A set of contigs corresponding to 1% of all contigs was randomly selected and used as reference. Intra-contig LDs within these contigs were performed for pairs of SNPs with minor allele frequencies (MAF) higher than 0.01.

### Identifying candidate genomic regions for selection in yam

We used four different approaches to identify regions under selection: two methods allowing identifying a reduction of diversity for the selected genes, two methods allowing identifying an excess of differentiation. The diversity reduction was assessed using Tajima’s D and by the ratio of cultivated to wild diversity. The excess of differentiation was assessed using the F_ST_ between cultivated and wild populations and a principal component based analysis. Tajima’s D value of each contig was calculated for the species using vcftools v0.1.13 [35]. (1) We plotted the distribution of Tajima’s D values and then used a 1% threshold to identify extremely low values. (2) The ratio of the cultivated genetic diversity divided by the mean diversity of the two wild relative species using π [29] and θ [30]. We used a 1% threshold to identify outlier contigs with extremely low ratios. (3) We estimated the differentiation index F_ST_ [36] between the cultivated group and each of the two wild groups for each contig using vcftools v0.1.13 [35]. Using the cutoff of the 1% top values, contigs with extreme F_ST_ between the cultivated and both two wild relatives were selected as candidates. (4) Based on principal component analysis at the SNP level we used the program Pcadapt V2.2 [37] to identify SNPs with extreme differentiation between the three species. The Mahalanobis distance [38] was calculated and we used the 5% threshold of the false discovery rate (FDR) [39] to detect candidate SNPs. The four selection tests were compared using a Venn diagram [40] to reveal the most likely candidate regions for selection. The annotation of the candidate selected genes was retrieved from a previous study [24].

### Enrichment analysis for annotated candidate contigs

First, all the candidate contigs annotated in the reference transcriptome were tested for enrichment of gene ontology (GO) molecular function terms. Standard Fisher’s exact tests implemented in the R package TopGO [41] were performed. A minimum of five annotated genes were required per term in order to limit statistical artifacts of GO terms with less annotated genes. Then, to control for false positive effects, only candidate contigs identified by at least two different selection tests were chosen, and the enrichment of GO terms analysis was rerun.

## Results

### Diversity structuration supports the three major species

We generated 162 million 100-bp paired-end reads. The yam transcriptome size has been estimated to be approximately 64 Mb [24] and the genome size to be 550 Mb. We obtained an average mapping rate of ~ 12.6% of our genomic reads i.e. close to the expected 12.4% based on the relative transcriptome size compared to the whole genome (Additional file 2: Table S2). We identified a total of 308,840 SNPs. These SNPs were found in 23,136 contigs with a mean contig length of 1316 bp (ranging from 250 to 15,691). A low correlation was observed between the length of the contigs and the number of SNPs detected (*r* = 0.34, *p* < 0.001)*.*

**Fig. 1:**
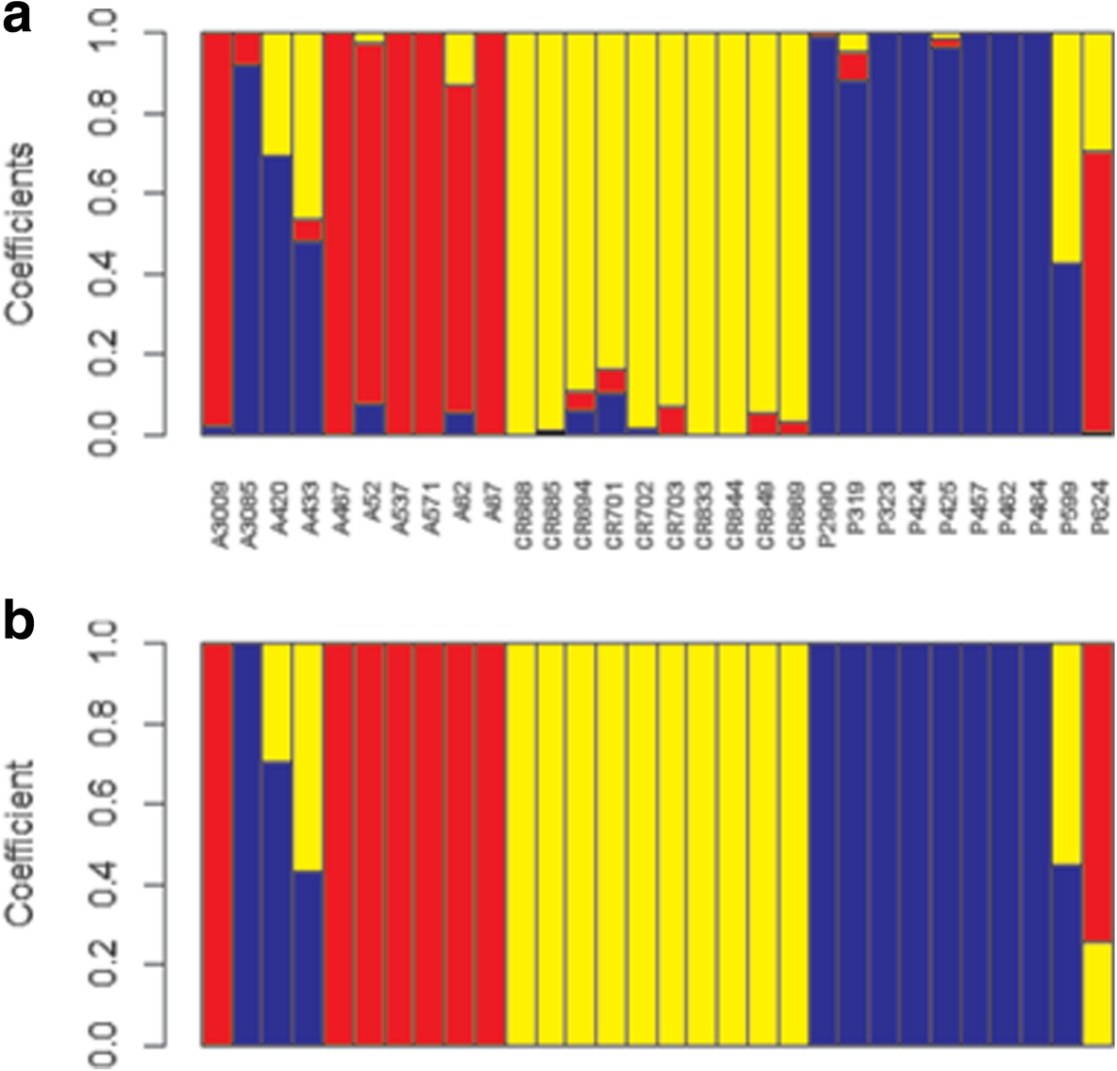
Structure analysis using sNMF(**a**) and NgsAdmix (**b**). Each color represents one population. The length of each segment in each vertical bar represents the proportion of ancestry in each population

Analysis of the population structure using sNMF led to three major genetic groups (Additional file 2: Figure S1), corresponding to the three species (Fig. 1-a). We identified four individuals (A420, P599, A433 and P624) as interspecific hybrids. One individual (A3085) was certainly misclassified in the field: it was recorded as *D. abyssinica* in the field but was genetically close to the *D. praehensilis* group. The exact structuration was similarly found using the NgsAdmix approach, with only minor differences in the estimated proportion of admixture (Fig. 1-b). As hybrids could bias the calculation of diversity; the differentiation tests; and Tajima’s D statistics, we removed the four hybrids for further analysis. Departures for neutrality or extreme differentiation were consequently assessed on 26 individuals.

We compared nucleotide diversity π and the nucleotide polymorphism θ between the cultivated species and each of the wild species. First, the cultivated diversity π was 26% and 36% respectively lower than *D. abyssinica* and *D. praehensilis* (Additional file 2: Table S3 a and b). Secondly, the cultivated diversity θ was 28% and 44% lower than *D. abyssinica* and *D. praehensilis* respectively. Linkage disequilibrium (LD) computed between 400,760 pairs of SNP decreased rapidly at *r* ^*2*^ = 0.1 after 100 bp (Additional file 2: Figure S2).

### The combination of selection tests identified a large set of candidate contigs

Contigs were searched for selection signatures using four different methods: Tajima’s D, marked reduction in the diversity in the cultivated samples, differentiation between wild and cultivated species, and principal component analysis. Using the four methods, a total of 998 candidate contigs were identified (Additional file 2: Table S4), among which 81 were detected by at least two methods (Additional file 2: Figure S3).

**Fig. 2:**
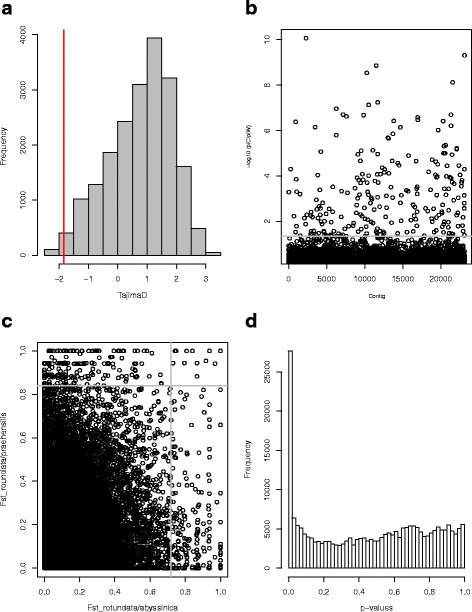
Summary of the different tests used to identify outlier contigs. In the distribution of Tajima’s D value of the cultivated species (**a**), the red line indicates the 1% threshold used to consider contigs as candidates. In the of reduction of nucleotide diversity π (**b**), the -log10 (π_c_/π_w_) for each contig is represented by one dot. The gray line corresponds to the 1% threshold used to consider contigs as candidates. In the comparison of F_ST_ between the cultivated and the two-wild species (**c**), each dot represents a contigs. The grey lines indicate the 1% threshold used to consider contigs as candidates. Finally, in the histogram of *p*-value (**d**), the peak of SNP close to zero indicates the presence of outliers. Here, the SNPs were considered as candidates using an FDR of 0.05

(i) Tajima’s D in the cultivated yam showed a skewed distribution to positive values (Fig. 2-a), with a mean of 0.77. The distribution reflected an excess of contigs with low diversity (Fig. 2-a). The distribution of Tajima’s values in the two wild species is centered on zero and consequently reflects a more global equilibrium between SNP occurrence and their frequencies (Additional file 2: Figure S4). Using a 1% threshold (Tajima D < −1.84), a total of 187 contigs were identified as potential candidates under selection in the cultivated sample.

(ii) The reduction of nucleotide diversity and the nucleotide polymorphism were highly correlated (*r* = 0.997, *p* < 0.001, (Additional file 2: Figure S5). Consequently, we only used the reduction of nucleotide diversity (π_c_/π_w_) for further analysis. Using a threshold of 1% (−log10 (π_c_/π_w_) > 1.34), a total of 232 contigs were identified as having an extremely low diversity in the cultivated sample compared to their wild relatives, and were therefore considered as candidates. (Fig. 2-b).

(iii) The average differentiation between *D. rotundata* and *D. praehensilis* was higher than between *D. rotundata* and *D. abyssinica,* (F_ST_ = 0.21 and 0.16, respectively, *p*-value <0.001). Using a 1% threshold (F_ST_ > 0.73 and 0.84 for *D. rotundata* with *D. praehensilis* and *D. abyssinica* respectively), 422 contigs were identified with extremely high F_ST_ values with one or the other wild species. Among them, 12 showed extreme values with the two wild species simultaneously (Fig. 2-c).

(iv) Last, we used a SNP-based approach. The two first principal components were used to perform the genome scan for selection using Pcadapt V.2.2 (Additional file 2: Figure S6a). The Mahalanobis statistic distance fitted a normal distribution (Additional file 2: Figure S6b). The histogram of *p*-values showed an excess of small p-values, indicating the presence of outliers (Fig. 2d). Using a 5% threshold, we identified 2502 SNPs in 1602 candidate contigs with extremely low p-values. A total of 238 contigs that showed at least two SNPs putatively under selection were retained as candidates.

### Root development, starch biosynthesis, phototropism and photosynthesis candidate genes were selected

**Fig. 3:**
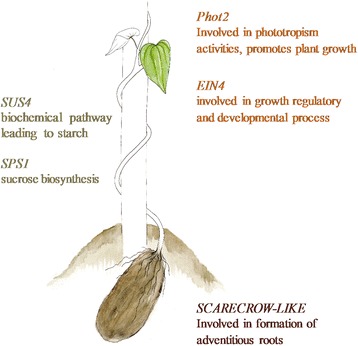
Key genes associated with yam domestication. *SCARECROW-LIKE, Phot2, EIN4, SUS4* and *SPS1* are some interesting genes probably selected during domestication

**Fig. 4:**
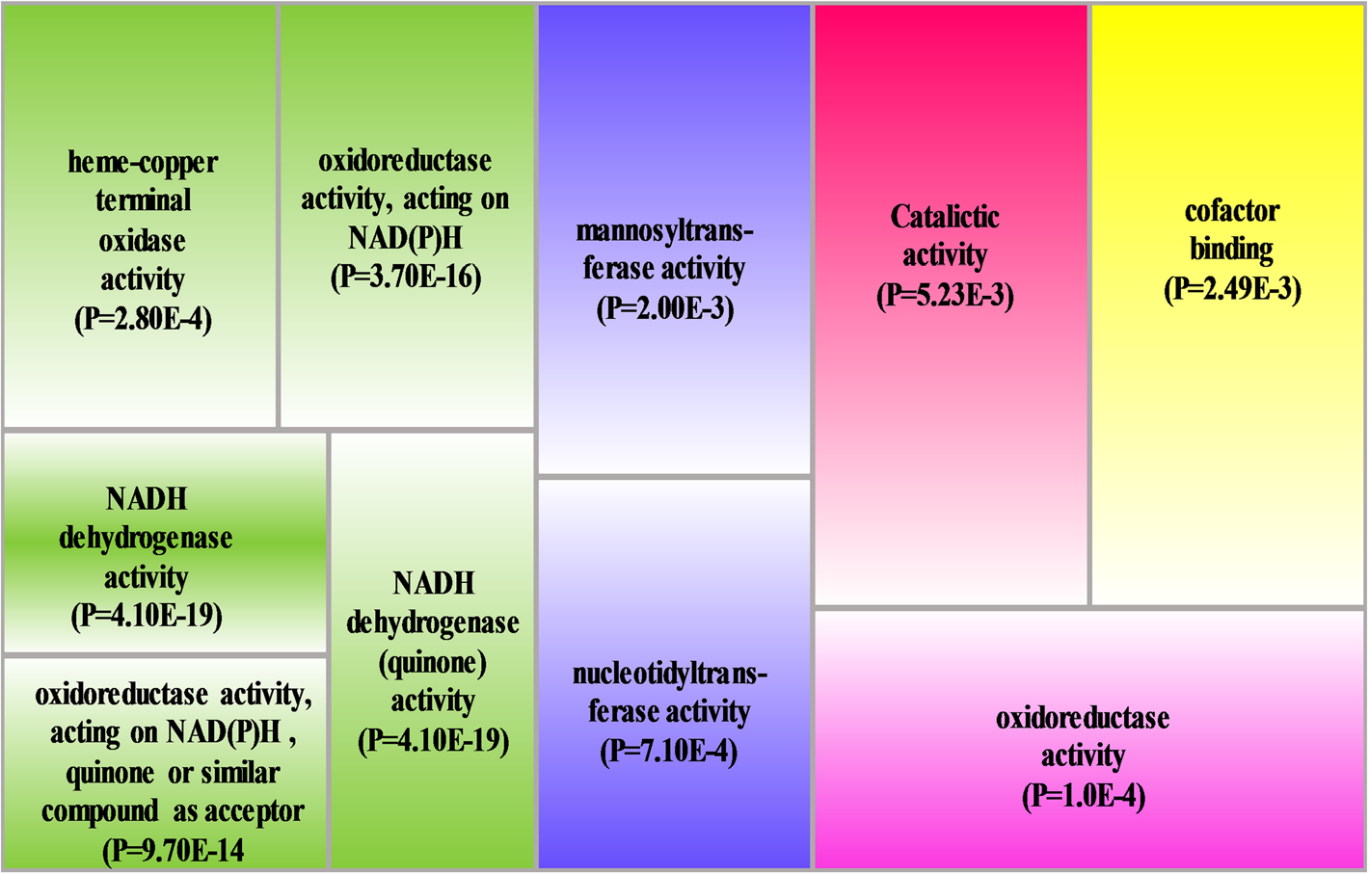
TreeMap view of the 10 most significant “Go Terms” identified. The 10 most significant GO terms were reported with their respective *p*-values. We group them in 4 major clusters: “oxidase activity” in green, “transferase activity” in blue, “catalytic activity” in pink. “cofactor binding” in yellow

We compared the candidate contigs with the available annotation of the yam transcriptome reference [24]. Thus, we retrieved some genes corresponding to putative targets for selection during yam domestication. In particular, among the genes annotated for the candidate genes, we identified five candidate contigs that were relevant in the light of yam domestication (Fig. 3 and Additional file 2: Table S5). These five candidate contigs showed strong diversity loss in the cultivated group compared to the wild species (Additional file 2: Figure S7). A candidate contig was a putative *SCARECROW-LIKE* gene involved in root development [42, 43]*.* Two other genes were associated with the earliest stages of starch biosynthesis and storage i.e., genes coding for the sucrose synthase 4 [44] and the sucrose-phosphate synthase 1 [45]. We also identified two genes associated with growth and phototropism, respectively*: Ethylene Insensitive 4* genes *(EIN4)* [46] and *Phototropin 2* gene (*Phot2,* [47]. The 998 candidate contigs were significantly enriched for a total of 21 significant GO terms (Additional file 2: Table S6). When we restricted our analysis to the 81 candidate contigs detected by at least two methods, we obtained nine significant GO terms (Additional file 2: Table S7). The most significant GO terms were identical whether we considered all the candidate contigs or only the 81 candidate contigs. The set of GO terms found across these two enrichment tests was associated with dehydrogenase and oxidoreductase (*NADH DH*) activities (Fig. 4).

## Discussion

### The domestication diversity loss observed in yam is comparable to an outcrossing crop

Today, the *D. rotundata* yam species is vegetatively propagated. However, the nucleotide diversity loss associated with domestication is relatively modest: the cultivated sample had 26% and 36% diversity loss respectively relative to *D. abyssinica* and *D. praehensilis*. In out-crossing species like pearl millet and maize, diversity losses of 32% [48] and 35% [49] were reported. In self-pollinating species, the diversity loss can be much higher, for example, 62% in barley [50], and 70% in wheat [51]. The loss of diversity observed in our study is more similar to outcrossing crops. We do not know when the transition from an outcrossing crop to a preferentially vegetative crop occurred. It is likely that during the first step of domestication, the crop reproduced mainly through seed. Even today, the reproduction system of *D. rotundata* is not purely vegetative [13, 52], and some cultivated varieties were found to have been recently obtained by cross-pollination. So, this modest loss of diversity is not surprising.

Linkage disequilibrium (LD) also decreased rapidly, like in other outcrossing crops. This LD decay is more similar to that observed in maize [53–55] than to that reported in self-pollinating crops such as rice [56]. However, our estimation of LD is based on a small sample and we might overestimate the rapidity of its decrease.

Overall, despite the mode of reproduction of the cultivated yam, both the diversity loss and the LD decay observed were similar to those in outcrossing crops.

### Identifying selected genes during domestication

We found 2% of yam genome classified as candidates for selected genes during domestication. A very similar rate of genome under selection was previously observed in maize, ranging from 2 to 5% [49, 57, 58]. Among the contigs we identified, roughly 10% of the candidate contigs were commonly identified by a least two different methods used for detecting signatures of selection.

Depending of the strength and the timing of selection, its resulting impact on diversity could differ. Consequently, each test has different strength and power to detect these specific signatures of selection. For example, when strongly selected, alleles could be fixed. These specific genes showing strong selection could be detected by differentiation F_ST_ based test, but not by Tajima’s D test because of their fixed polymorphism [31]. So, the specificity of each test could lead to the discovery of only a small set of the same contigs by all different methods. However, each method could also identify false positives [59]. These false positives could be specific of a test. In conclusion, both false positives and different impacts of selection on diversity resulted in roughly 10% of genes being simultaneously identified by all the methods performed. Furthermore, signature of selection on two contigs could be associated with a single selection events one of them. Even if we found that linkage disequilibrium decreased fast, our list of selected genes might represent fewer selection events than their actual numbers.

### Domestication is associated with selection of root development, sugar metabolism, and phototropism genes

Cultivated yams are known to have less ramified and larger roots than wild yams. Remarkably, we found a contig homologous to a gene coding for a *SCARECROW-LIKE* protein. As demonstrated in *Arabidopsis*, this gene is a key player in root development [42, 43] and consequently may have been mobilized during yam domestication. We also pinpointed a contig homologous to an *EIN4* gene. *EIN4* is a receptor of ethylene [46] involved in growth regulation and many developmental processes including seed germination, leaf and flower senescence [60]. At this stage, we do not know if this gene may affect root development itself or its above ground development.

Domestication of root and cereal crops is notably associated with the increase of starch production. Several studies on cereals suggest that starch biosynthesis and storage were important targets for selection [61]. In our study, we observed the selection of two genes involved in the production of sugar: *SUS*4 and *SPS1*. *SUS* catalysis is the first step leading to starch formation [44] by converting sucrose to fructose and UDP-glucose. In wheat, selection for increased starch content was associated with selection of *SUS* genes [62], and enhancing *SUS* activities also resulted in increasing starch content in maize [63]. The *SPS* gene has also been reported to play a major role in sucrose biosynthesis under osmotic stress conditions [45]. In conclusion, similar set of genes were selected during cereal, root and tuber crops.

Beyond starch production, cultivated yam underwent a major change in its living environment during domestication. Yams are now grown in open fields, whereas its wild relatives grow as vines in the shade of tutor trees. This environmental change during domestication certainly required adaptation due to such changes in light and heat. We observed strong signatures of selection in genes associated with physiological processes of regulation of photosynthesis for light tracking and for plant growth. Indeed, one of our candidate contigs is homologous to the *Phototropin 2* gene (*Phot2*). In higher plants, Phot2 enables perception of blue light and consequently optimization of photosynthetic performance and growth [47].

### Adaptation to high intensity light was selected during yam domestication

Beyond specific genes associated with the change from shade to light environment, we also found a significant enrichment of interesting gene ontology terms. The most significant GO terms observed were and oxidoreductase activities associated with *NADPH DH* complex genes [64, 65]. Whatever the strategy of enrichment test used, the results were robust for these functions. The *NADH DH* complex is an important set of enzymes for chlororespiration [66]. The *NADH DH* complex is involved in photosynthesis [67], more specifically in the photosystems I (PSI) and II (PSII). It plays a role in protection against photo-oxidative stresses associated with the formation of reactive oxygen species (ROS) [68]. High light and heat could favour the production of ROS [69, 70]. In oats, NADH DH is over-expressed with increasing light [67]. Consequently, it has been postulated that this type of complex plays a role in mitigating ROS stress associated with increasing intensity of light or heat. In Brassica plants, the same *NADH DH* complex has also been reported to be associated with the domestication process [71]. The wild species of *Brassica* showed higher tolerance to high light and heat intensity than the cultivated species [71]. In this specific case, domestication was associated with a decrease in photosynthetic parameters under stress conditions in the cultivated species [71]. The two wild species of yam are vines that grow in partial shade. The cultivated species *D. rotundata* grows under full sunlight in the field. We hypothesize that adaptation of the cultivated yam led to the selection of genes that enable efficient photosynthesis with increasing light and heat intensity. Optimizing photosynthesis is also an important way to enhance production of carbohydrate, later stored as starch in the tuber.

## Conclusions

Selection in the early step of sugar biosynthesis is detected in yam, and previously detected in cereal. This result suggests that key step in starch biosynthesis were necessary both in cereal as well as in root and tuber crops. More interestingly, drastic changes in habitat associated with domestication is certainly retraced in selection in phototropism genes. Selection on dehydrogenase and oxidoreductase activities associated with *NADPH DH* complex genes, was certainly the consequence of adaptation to optimize photosynthesis in full light. If some convergence is observed at the molecular level, very specific adaptations were necessary for the domestication of African yam. Beyond domestication, this study highlight the molecular mechanism associated with changes from shade-tolerant plant to a full light environment.

## Additional files

Additional file 1: We assess if the mapping of genomic DNA reads on a transcriptome reference could impact SNP calling in our special case. **Table S1.** Summary of mapping and SNP calling using simulated data. (DOCX 15 kb)

Additional file 2: Molecular basis of African yam domestication: analyses of selection point to starch biosynthesis, root development and photosynthesis related genes. **Table S1.** Passport data of plant material collected from Benin. **Table S2.** Metric information of data filtering and mapping. **Table S3.** Mean Nucleotide diversity (π) and polymorphism (ɵ). **Table S4.** List of the contigs detected as selected by at least one method. **Table S5.** Remarkable candidate genes showing selection signature. **Table S6.** Gene Ontology (GO) terms significantly enriched (*p*-value ≤ 0.05) among the 998 candidate contigs. **Table S7.** Gene Ontology (GO) terms significantly enriched (*p*-value ≤ 0.05) among the 81 candidates contigs detected by a least two methods. **Figure S1.** Cross-entropy calculated using sNMF (Frichot et al., 2014) for K = 1 to 6. Ten repetitions of the run were done. **Figure S2.** Intra-contigs linkage disequilibrium (LD) as a function of physical distance between SNPs pairs from 1% of all contigs. **Figure S3.** Venn Diagram comparing the candidate contigs obtained using the 4 methods. **Figure S4.** Distribution of Tajima’s D value calculated for *D. abyssinica* (a) and *D. praehensilis* (b). **Figure S5.** Comparison of diversity lost. **Figure S6.** Variance explained by PCA axis (a) and distribution of Mahalanobis distance (b) from PCAdapt. **Figure S7.** Nucleotide diversity within five candidate contigs for cultivated and the wild species (XLSX 45 kb)

## Abbreviations

BWA: Burrows-wheeler aligner
GATK: Genome analysis tool kit
GeT: Genome and transcriptome
LD: Linkage disequilibrium
LDcorSV: Linkage disequilibrium corrected by the structure and the relatedness
SOAP: Short oligonucleotide analysis package
SPS: Sucrose-phosphate synthase
SUS: Sucrose synthase
