# Supplementary material for: Molecular basis of African yam domestication: analyses of selection point to root development, starch biosynthesis, and photosynthesis related genes
Source: BMC Genomics. 2017 Oct 12;18:782. doi: 10.1186/s12864-017-4143-2 (PMC5639766; doi:10.1186/s12864-017-4143-2)
Supplement: Supplementary file 1 — We assess if the mapping of genomic DNA reads on a transcriptome reference could impact SNP calling in our special case. Table S1. Summary of mapping and SNP calling using simulated data. (DOCX 15 kb) [file 12864_2017_4143_MOESM1_ESM.docx]

***Supplementary file***

We assess if the mapping of genomic DNA reads on a transcriptome reference could impact SNP calling in our special case.

**Material and Methods.**

We simulated a 100kb reference genome. From this genome, using BedTools (Quinlan and Hall, 2010) we deleted sequence in intervals of 400bp corresponding to introns, to simulate a transcriptome reference. The 400bp deletions were closed to what is observed rice (<http://rice.plantbiology.msu.edu/analyses_facts.shtml>).

Using wgsim (<https://github.com/lh3/wgsim>), we simulated synthetic paired-end reads based on the normal 100kb reference reads with an expected coverage of 30x. Wgsim was set for reads length=150, number of reads=10,000, the base quality was set at 30 and we used the option –e0.001 for allowing sequencing error. Finally, we simulated the mapping of genomic DNA reads on a transcriptom reference to validate our approach. We used default option of bwa aln-sampe to map our reads on the two reference genomes i.e. the normal reference and the truncated one. In each case, we used GATK HaplotypeCaller (Citation) to call SNPs variant.

**Simulating mapping and SNP calling using synthetic data**

The truncated reference lead to less read mapped as expected: roughly 35% of the read mapped (Table 1). But, whatever the reference used, i.e. the normal genome of reference and the truncated one, we did not call more variant in the truncated version or the normal version (Table 1). So we do have major issue of our mapping strategy. We might however, perhaps detected some effect if sequencing depth is low.

**Table 1.** Summary of mapping and SNP calling using simulated data

|  |  | Number of mapped reads | | Number of SNPs | |
| --- | --- | --- | --- | --- | --- |
| ID_name | Total_Reads | Normal_Ref | Truncated_Ref | Normal_Ref | Truncated_Ref |
| ID1 | 20000 | 20000 | 6836 | 0 | 0 |
| ID2 | 20000 | 20000 | 7062 | 0 | 0 |

**Reference:**

McKenna A, Hanna M, Banks E, Sivachenko A, Cibulskis K, Kernytsky A, Garimella K, Altshuler D, Gabriel S, Daly M, DePristo MA. 2010. [The Genome Analysis Toolkit: a MapReduce framework for analyzing next-generation DNA sequencing data.](https://www.ncbi.nlm.nih.gov/pubmed/20644199) Genome Res. 20(9):1297-303.

[Quinlan AR](https://www.ncbi.nlm.nih.gov/pubmed/?term=Quinlan%20AR%5BAuthor%5D&cauthor=true&cauthor_uid=20110278), [Hall IM](https://www.ncbi.nlm.nih.gov/pubmed/?term=Hall%20IM%5BAuthor%5D&cauthor=true&cauthor_uid=20110278). 2010. BEDTools: a flexible suite of utilities for comparing genomic features. Bioinformatics 26(6):841-842.
